# Influence of Viscose Fibre Geometry on the Structure–Property Relationships of High-Density Polyethylene Composites

**DOI:** 10.3390/polym14204389

**Published:** 2022-10-18

**Authors:** Janez Slapnik, Gregor Kraft, Thomas Wilhelm, Marcel Hribernik, Iztok Švab, Thomas Lucyshyn, Gerald Pinter

**Affiliations:** 1 Faculty of Polymer Technology, Ozare 19, 2380 Slovenj Gradec, Slovenia; 2 Department of Polymer Engineering and Science, Polymer Processing, Montanuniversitaet Leoben, Otto Gloeckel-Strasse 2, 8700 Leoben, Austria

**Keywords:** thermoplastic matrix composites, high-density polyethylene, renewable fibres, man-made cellulose fibres, fibre geometry, compounding, injection moulding, microstructure, fibre length, mechanical properties

## Abstract

This study investigated the influence of viscose fibre (VF) geometry on the microstructures and resulting properties of high-density polyethylene (HDPE) composites. Seven types of viscose fibres varying in cross-section shape, linear density, and length were pelletised, compounded into HDPE with a twin-screw extruder, and injection moulded. The microstructures of the composites were characterised by investigating their cross-sections and by extracting the fibres and measuring their lengths using optical microscopy (OM). The mechanical and thermal properties of the composites were characterised using differential scanning calorimetry (DSC), tensile tests, Charpy impact tests, and dynamic mechanical analysis (DMA). The composites prepared using cylindrical fibres with a linear density of 1.7 dtex exhibited the best fibre dispersion, highest orientation, and lowest fibre–fibre contact area. The decrease in the linear density of the cylindrical fibres resulted in increasingly worse dispersion and orientation, while composites containing non-cylindrical fibres exhibited a comparably larger fibre–fibre contact area. The initial fibre length of about 3 to 10 mm decreased to the mean values of 0.29 mm to 0.41 mm during processing, depending on the initial geometry. In general, cylindrical fibres exhibited a superior reinforcing effect in comparison to non-cylindrical fibres. The composites containing cylindrical fibres with a linear density of 1.7 dtex and a length of 5 mm exhibited the best reinforcing effect with an increase in tensile modulus and strength of 323% and 141%, respectively.

## 1. Introduction

In recent years, thermoplastic matrix composites (TMCs) reinforced with renewable fibres (RFs) gained significant attention from academia and industry due to their potential to substitute conventional TMCs (e.g., glass fibre (GF)-reinforced) while being more sustainable. Lignocellulosic and cellulosic fibres, such as natural fibres (NFs) or man-made cellulose fibres (MMCFs), are especially attractive renewable reinforcements due to their large abundance, high specific mechanical strength, and relatively high thermal stability. Often, they are blended into thermoplastic matrices, such as HDPE, polypropylene (PP), and polylactic acid (PLA), by a twin-screw extruder and subsequently processed into final parts by injection moulding. Such composites can offer several advantages in comparison to their conventional counterparts that include a lower depletion of non-renewable resources, lower CO_2_ footprint, lower density, less abrasion to processing equipment, and less burdensome waste disposal. However, the successful preparation of an RF-reinforced TMC that satisfies increasingly demanding applications has proven challenging. First, RF possesses inherent limitations for use in TMCs, such as high moisture uptake, poor compatibility with non-polar matrices, limited thermal stability, and low bulk density, which results in challenging dosing during processing [1,2,3,4,5,6]. The latter limitation is often overcome by compressing the fibres into pellets, which solves the dosing issues but results in decreased dispersion and fibre damage [5,7,8,9]. Second, there are concerns about ensuring a consistent quality of composites due to year-to-year fluctuation in the properties of renewable materials and emissions of volatile organic compounds (VOC) in final applications [10,11]. In this regard, MMCFs offer significant advantages in comparison to NFs as their production is subjected to quality assurance protocols and due to their chemical purity. Furthermore, their production process enables obtaining fibres of different geometries (cross-section shape, linear density, and length) that can be precisely defined and consistent [12]. The mechanical properties of RF-reinforced TMCs depend on the properties of the matrix and fibres, fibre volume fraction, matrix–fibre interface, fibre aspect ratio (AR), and various microstructural factors such as the fibre orientation and fibre–fibre contact area [3,13,14]. In addition, unlike GF or carbon fibre, NF or MMCF tends to bend, twist, or even entangle in the polymer matrix, which impedes the effective stress transfer to the fibres and reduces the composites’ mechanical properties [15]. A large number of studies concerning RF-reinforced TMCs have investigated the influence of different matrix/fibre combinations [3], fibre loadings [16], processing parameters [17,18,19], and strategies for improving matrix–fibre interactions [20,21], but fewer have systematically investigated the influence of fibre geometry, which is also known to have a great impact on the final properties. In general, the optimal mechanical properties of TMCs are obtained when fibres exceed the critical aspect ratio, which marks the point above which a sufficient amount of stress can be transferred to the fibres which result in fibre breakage during composite loading [14]. However, using fibres with a high initial AR does not guarantee the good performance of NF- or MMCF-reinforced composites. In contrast, several studies have shown that a higher initial fibre AR led to decreased mechanical properties, which could result from higher fibre breakage or poor fibre dispersion and orientation [22,23,24,25]. The fibre cross-section shape can also have an important influence on the properties of composites, but this area is even less explored, especially for TMCs. It has been shown that for unidirectional GF-reinforced thermoset composites, a higher fibre cross-sectional aspect ratio can lead to higher toughness, while a triangular cross-section can lead to higher tensile and compression strength in comparison to conventional cylindrical fibres [26,27,28].

The present study aimed to evaluate how VF geometry influences the mechanical and thermal properties of TMCs. Furthermore, the goal was to investigate the microstructure of the composites and explain the relationships between the fibre geometry, microstructure of the composites, and resulting properties. In this way, the results of the present study should provide better insights and guidelines for further optimisation of the properties of TMCs reinforced with MMCF or NF. HDPE was chosen as a polymer matrix due to its relatively low processing temperature, which limits the thermal degradation of VF. Seven different types of VFs were used that varied in their geometry. Four types of fibres had a circular cross-section and varied in linear density and length, two had rectangular cross-sections of different aspect ratios, while one fibre had a trilobal cross-section.

## 2. Materials and Methods

### 2.1. Materials

The polymer matrix was HDPE CC2056 (SABIC, Riyadh, Saudi Arabia). HDPE grafted with maleic anhydride (HDPE-g-MA) Exxelor^®^ PE 1040 (ExxonMobil, Irving, TX, USA) was used as a compatibiliser. Erucamide Crodamide ER (Croda, Snaith, United Kingdom) was used as a slip agent. Pentaerythritol tetrakis(3,5-di-tert-butyl-4-hydroxyhydrocinnamate) AT10 (Amik Italia S.p.A., Milano, Italy) was used as an antioxidant. VFs were kindly provided by Kelheim Fibres GmbH (Kelheim, Germany). Seven different samples of VFs were used in the study, with their commercial names, geometrical properties, and abbreviations of the corresponding composites summarised in Table 1. The concept of equivalent radius that transforms fibres with a non-circular cross-section shape into the cylindrical fibre of equivalent cross-section surface area was used to determine an equivalent diameter (*D*_EQ_) to estimate the fibre-reinforcing potential [13]. *D*_EQ_ was calculated using fibre linear densities provided by the manufacturer and a density of 1.52 g/cm^3^.

### 2.2. Sample Preparation

#### 2.2.1. Pelletising

The fibres were pelletised using a PTA 50 (Tecno Aspira, Novedrate, Italy) pelletiser. The pelletiser had a 4 kW electric motor and a 6 mm die plate. A slip agent (60 g on 1 kg dry mass of VF) was added to the fibres by dry mixing to decrease the friction during the pelletising process and to enhance fibre dispersion into the polymer matrix. Before pelletising, the fibres were conditioned to a moisture content of 20 wt.%.

#### 2.2.2. Compounding

The VF pellets were dried prior to processing in a laboratory oven 100–800 (Memmert, Büchenbach, Germany) at 105 °C to below a moisture content of 0.03 wt.% and mixed by hand with HDPE granules and other additives to obtain the composition presented in Table 2. Composites were prepared by compounding using a co-rotating twin-screw extruder LTE 20–44 (Labtech Engineering, Samut Prakan, Thailand). The extruder had a screw diameter of 20 mm and an *L*:*D* ratio of 44:1. The extrusion temperature profile is presented in Table 3. The screw speed was 400 min^−1^. The extruded filaments were cooled in a water bath and granulated using a Scheer SGS 25-E4 (Maag Pump Systems AG, Oberglatt, Switzerland) to 10 mm long granules.

#### 2.2.3. Injection Moulding

Composites were dried prior to processing in a 100–800 (Memmert, Büchenbach, Germany) laboratory oven at 105 °C to a moisture content of below 0.03 wt.%. Test specimens according to ISO 527-2 (type 1BA) and ISO 179-1 were injection moulded using a CX 50-180 (Krauss-Maffei, München, Germany) injection-moulding machine with a screw diameter of 30 mm, an *L*:*D* ratio of 23.3:1, and a maximum clamping force of 500 kN. Injection moulding processing parameters are summarised in Table 4.

### 2.3. Characterisation

#### 2.3.1. Optical Microscopy

Optical micrographs were captured using a VHX-7000 (Keyence, Osaka, Japan) digital microscope with a VH-ZST lens. Fibre dispersion and distribution were evaluated by preparing approximately 20 µm thick slices of cross-sections of tensile test specimens using a microtome and capturing micrographs using a transmitted illumination at 300× magnification.

#### 2.3.2. Fibre Length

Fibres were extracted by boiling Charpy impact specimens in xylene under reflux for 10 h, filtering the extracted fibres under vacuum, and rinsing the fibres with boiling xylene to remove residual HDPE. The resulting fibres were dispersed in water and captured using an LCD Micro (Bresser, Rhede, Germany) at 4× magnification. Fibre length was determined by tracing 500 fibres with polylines along their lengths in ImageJ 1.53c software.

#### 2.3.3. Differential Scanning Calorimetry

Thermal properties were determined using a DSC 2 (Mettler Toledo, Greifensee, Switzerland) calorimeter in 40 µL aluminium crucibles. Samples were tested in a temperature range of 20 °C to 170 °C, with a heating/cooling rate of 10 K/min in a nitrogen (N_2_) atmosphere (20 mL/min). Isothermal segments before heating and cooling segments were set to 5 min. The degree of crystallinity (*X*_c_) was calculated according to the following equation:

*X*_c_ = Δ*H*_m_/(*w*_PE_ × Δ*H*_0_) × 100% (1)

where Δ*H*_m_ is the sample melting enthalpy, *w*_PE_ is the mass fraction of HDPE and HDPE-g-MA in the sample, and Δ*H*_0_ is the melting enthalpy of 100% crystalline HDPE (293 J/g) [29].

#### 2.3.4. Tensile Tests

Tensile properties were determined using an Ag-X plus 10 kN (Shimadzu, Kyoto, Japan) universal testing machine according to ISO 527-1 standard. The gauge length was 50 mm, preload was 3 N, and testing speed was 1 mm/min until 0.25% strain and 50 mm/min until breaking. The results are average values of five measurements.

#### 2.3.5. Charpy Impact Tests

The Charpy impact strength was determined using an LY-XJJD5 (LIYI, Dongguan, China) pendulum impact tester, according to ISO 179-1 standard. Notched and unnotched specimens were measured using 2 J and 5 J pendulums, respectively. The results are average values of 10 measurements.

#### 2.3.6. Dynamic Mechanical Analysis

Dynamic mechanical properties were determined using a DMA 8000 (Perkin Elmer, Waltham, MA, USA) dynamic mechanical analyser according to ASTM D5418 standard. Samples were tested in flexure using dual cantilever beam supports. Samples were heated from 28 °C to 130 °C, with a heating rate of 2 K/min. The frequency was 1 Hz, and the amplitude was 0.02 mm.

## 3. Results

### 3.1. Optical Microscopy

**Figure 1:**
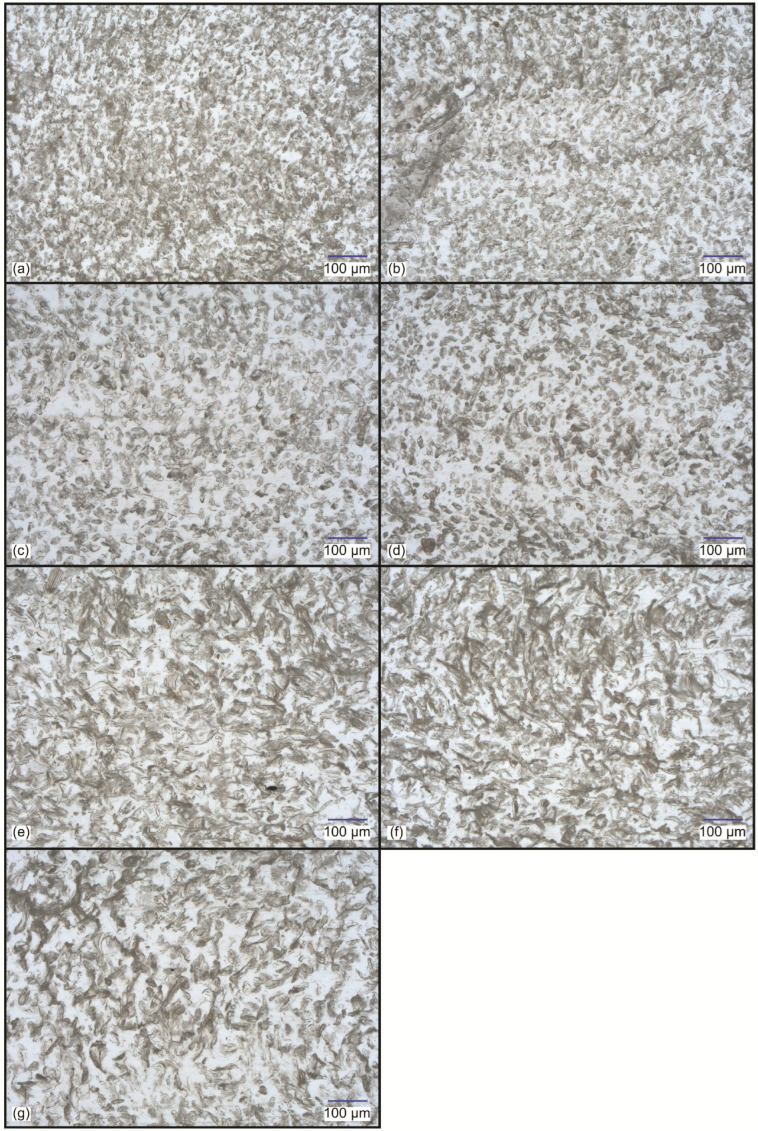
Optical micrographs of tensile test specimens’ cross-sections of samples: (**a**) D_0.5-3; (**b**) D_0.9-5; (**c**) D_1.7-5; (**d**) D_1.7-10; (**e**) V_2.4-5; (**f**) L_2.5-5; (**g**) G_3.3-5.

Optical micrographs of microtome-cut cross-sections of the tensile test specimens of the different samples are presented in Figure 1. Samples D_1.7-5 (Figure 1c) and D_1.7-10 (Figure 1d) had a similar microstructure with fibres randomly distributed and relatively well dispersed. A majority of fibres were oriented completely parallel to the flow direction. A decrease in the fibre linear density (samples D_0.9-5 and D_0.5-3 in Figure 1a,b, respectively) resulted in similar fibre distribution but increasingly worse fibre dispersion and a larger fibre–fibre contact area. It appears that a decrease in linear density also resulted in a more random fibre orientation, which may be attributed to higher fibre agglomeration. Samples V_2.4-5 (Figure 1e) and L_2.5-5 (Figure 1f) had similar microstructures with fibres randomly distributed but with a seemingly large fibre–fibre contact area. A large portion of fibres was oriented parallel to the flow direction, albeit the orientation was somewhat more random in comparison to the cylindrical fibres with a linear density of 1.7 dtex. It seems that processing damaged a large portion of the Viloft^®^ and Leonardo^®^ fibres by splitting them along their lengths and decreasing their widths. Despite that, the higher cross-sectional aspect ratio of the latter fibres was still well visible in the micrographs. Sample G_3.3-5 (Figure 1g) was prepared using trilobal-shaped fibres. However, the fibres were severely damaged during the processing, resulting in a complete deterioration of their cross-sectional shape into smaller pieces of random shapes. Overall, the composites had a similar random distribution of the fibres but significantly differed in terms of fibre dispersion, fibre–fibre contact area, and fibre orientation. It appears that the composites prepared from cylindrical fibres with a linear density of 1.7 dtex had a microstructure with the best fibre dispersion, lowest fibre–fibre contact area, and highest fibre orientation. The decrease in the fibre linear density resulted in increasingly worse fibre dispersion and orientation. Non-cylindrical fibres were relatively well distributed, dispersed, and oriented. However, their fibre–fibre contact area seems to be comparably large, which can be explained by the different packing properties of fibres with circular and rectangular cross-sections. In the maximum packing configuration of cylindrical fibres, a large portion of the fibre surface area is still surrounded by the matrix. In contrast, fibres with rectangular shapes can be packed in a configuration where fibres are completely in contact with each other.

### 3.2. Fibre Length

**Figure 2:**
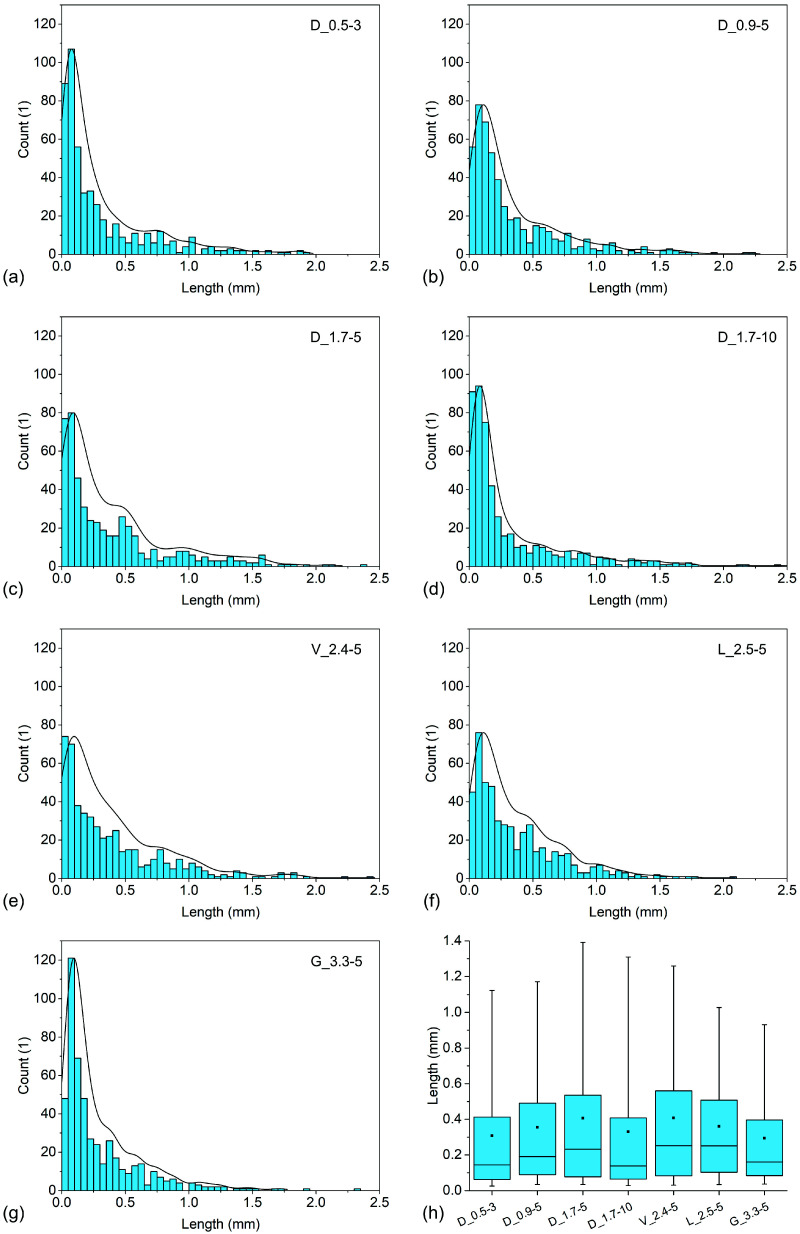
Fibre length distribution representations in Charpy impact test specimens of samples: (**a**) D_0.5-3; (**b**) D_0.9-5; (**c**) D_1.7-5; (**d**) D_1.7-10; (**e**) V_2.4-5; (**f**) L_2.5-5; (**g**) G_3.3-5; and (**h**) comparison of all samples.

The fibre length distribution of the samples is presented in Figure 2. Whiskers on the box plots represent the 5th and 95th percentiles while black squares represent arithmetic mean values. All samples contained significantly shorter fibres in comparison to their initial length and exhibited a right-skewed fibre length distribution. Sample D_1.7-5 contained the longest fibres (mean of 0.41 mm, a median of 0.23 mm) and exhibited the widest distribution of fibre length among the samples containing cylindrical fibres, with upper and lower quartile values of 0.54 mm and 0.08 mm, respectively. Increasing the initial fibre length from 5 mm to 10 mm (sample D_1.7-10) resulted in a significant decrease in fibre length (mean of 0.33 mm), with half of the fibres being less than 0.14 mm long, while the upper and lower quartile values decreased to 0.41 mm and 0.06 mm, respectively. Decreasing linear density to 0.9 dtex had a similar effect but to a lesser extent as the fibre length decreased to a mean of 0.36 mm and a median of 0.19 mm. The upper quartile value of sample D_0.9-5 (0.49 mm) was lower in comparison to sample D_1.7-5, while the lower quartile value was slightly higher (0.09 mm). Sample D_0.5-3 contained fibres of similar length as sample D_1.7_10, with the upper quartile, mean, median, and lower quartile values of 0.41 mm, 0.31 mm, 0.14 mm, and 0.06 mm, respectively. However, for this sample it is not possible to reliably estimate which factor contributed to the low fibre length as both the fibre linear density and initial fibre length differed from the rest of the tested samples. Sample V_2.4-5 had the highest upper quartile (0.56 mm), mean (0.41 mm), and median (0.25 mm) values of fibre length among the non-cylindrical fibres, with a relatively similar fibre length distribution to sample D_1.7-5. In comparison, sample L_2.5-5 had a slightly lower upper quartile (0.51 mm) and mean (0.36 mm), with a comparable median value (0.25 mm) and higher lower quartile (0.10 mm) values of fibre length. Sample G_3.3-5 contained the shortest fibres among the non-cylindrical fibres, with upper quartile (0.40 mm), mean (0.29 mm) and median (0.16 mm) values of fibre length, while the lower quartile (0.08 mm) value was comparable to sample L_2.5-5. Regarding cylindrical fibres, the results of the study indicate that an optimal initial viscose fibre linear density and length exist in terms of preserving their length during processing. A higher initial fibre length can lead to a decreased fibre length in the composites, which can be explained by the higher tendency of longer fibres to entangle and consequently higher fibre damage during processing [30]. The opposite trends were noticed in terms of fibre linear density, where lower values resulted in shorter fibres. Similarly, this can be explained by the higher tendency of thinner fibres to entangle due to a less stiff structure in combination with stronger inter-fibre physical interactions (e.g., hydrogen bonds) resulting from the higher specific surface area [30,31]. This effect should also be responsible for poor dispersion and orientation of the thinner fibres in the composites. Fibre cross-section also plays an important role in terms of the shortening of fibres during processing. Viloft^®^ and Leonardo^®^ fibres had the same length and similar linear densities, but the latter fibres had a much higher cross-section aspect ratio, which resulted in a lower fibre length. This effect may be attributed to higher inter-fibre forces due to larger contact areas between the fibres and lower thickness, which makes fibres harder to disperse and easier to break. Galaxy^®^ fibres were severely shortened during the processing, which may be caused by the mechanical interlocking of the fibres in the pellets due to their trilobal shape that impeded the dispersion and resulted in high fibre damage. Furthermore, their sides are relatively thin and can easily break during processing. A very important consideration is that the fibres were first compressed by a pelletising process, which makes compounding significantly less challenging and more consistent but is known to result in fibre damage and impede fibre dispersion [5,7,8,9]. Thus, the results may have been different if the composites were produced from uncompressed pellets fed using a suitable dosing system.

**Figure 3:**
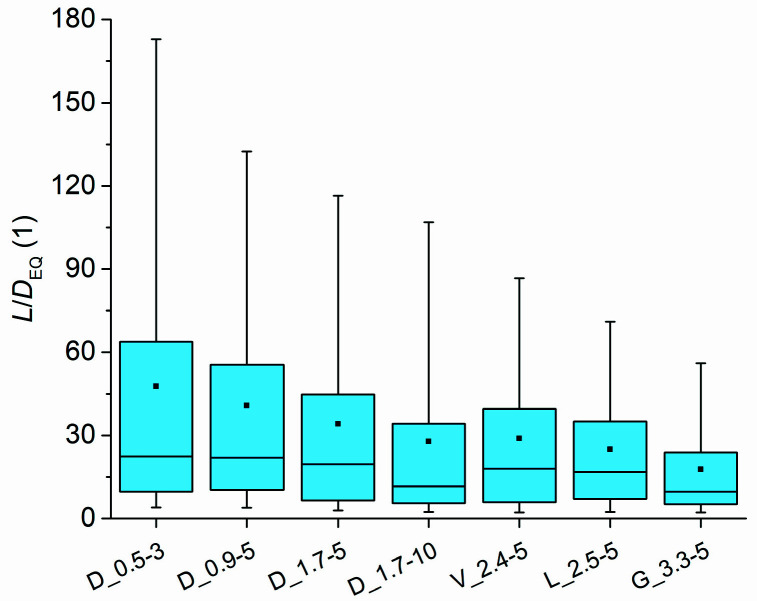
Estimated distribution of fibre aspect ratio in the composites.

The fibre aspect ratio of the composites was estimated by dividing the measured fibre length (*L*) by the *D*_EQ_ value. The estimated aspect ratio (*L*/*D*_EQ_) distributions of the fibres in the composites are presented in Figure 3. Sample D_0.5-3 contained fibres with the highest aspect ratio with upper quartile, mean, median, and lower quartile values of 64, 48, 22, and 10, respectively, despite the fibres having the lowest length among the cylindrical fibres. Sample D_0.9-5 contained fibres of lower upper quartile (55) and mean (41) values of aspect ratio, while the median and lower quartile values were approximately in the same range. Sample D_1.7-5 contained fibres with a significantly lower aspect ratio with upper quartile, mean, median, and lower quartile values of 45, 34, 20, and 7, respectively. Sample D_1.7-10, which was produced using fibres with the highest initial aspect ratio (838), had fibres with the lowest aspect ratio among the composites containing cylindrical fibres, with upper quartile, mean, median, and lower quartile values of 34, 28, 12, and 5, respectively. Micrographs of the cross-sections of the tensile test specimens revealed that the non-cylindrical fibres were significantly damaged during processing and some portions of the fibres were split along their lengths, resulting in a smaller cross-section area. Therefore, it is assumed that the *L*/*D*_EQ_ values of the non-cylindrical fibres were slightly underrepresented, especially for Galaxy^®^ fibres. Nevertheless, the results should still provide some insight into the approximate aspect ratio of the fibres. Among the latter composites, sample V_2.4-5 contained fibres with the highest upper quartile (40), mean (29), and median (18) values of aspect ratio, while sample L_2.5-5 contained fibres with slightly higher lower quartile (7) but lower upper quartile (35), mean (25), and median (17) values of aspect ratio. Sample G_3.3_5 contained fibres with by far the lowest aspect ratio, with upper quartile, mean, median, and lower quartile values of 24, 18, 10, and 5, respectively.

### 3.3. Differential Scanning Calorimetry

**Figure 4:**
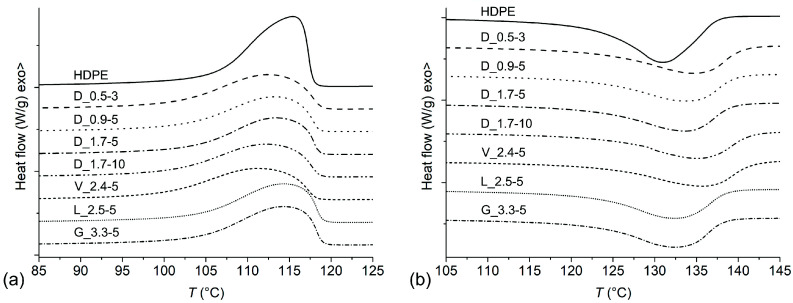
DSC thermograms of samples: (**a**) first cooling run; (**b**) second heating run.

DSC thermograms of the first cooling run and second heating run are presented in Figure 4a,b, respectively, while the corresponding thermal properties are presented in Table 5. In comparison to neat HDPE, the composites exhibited an increased onset crystallisation temperature (*T*_c,onset_) by up to 1.1 K, a decreased crystallisation temperature (*T*_c_) by 1.1 K–4.2 K, and an increased crystallisation peak width (*T*_c,width_) by 2.0 K–5.0 K. The increased *T*_c,onset_ of the composites suggests that VF and/or additives promote heterogeneous nucleation of HDPE, while the decreased *T*_c_ and increased *T*_c,width_ were attributed to steric hindering of the crystallisation by the VF in combination with a decreased crystallisation rate due to the presence of amphiphilic erucamide [32,33]. These phenomena resulted in the growth of larger and less uniform crystallites, which was reflected in an increased melting temperature (*T*_m_) by 2.7 K–4.6 K and increased melting peak width (*T*_m,width_) by 1.2 K–2.7 K. The decrease in *T*_c_ and increase in *T*_m_ of HDPE by the addition of VF were also reported by other authors [34]. The composites exhibited lower enthalpies of crystallisation (Δ*H*_c_) and melting (Δ*H*_m_) in comparison to neat HDPE by 52.1 J/g–56.8 J/g, which equates to slightly higher estimated crystallisation degrees of the contained HDPE (up to 2.5%). Similar estimated crystallisation degrees of neat HDPE and composites indicate accurate fibre loadings in the composites. It was found that *T*_c,width_, *T*_m_, and *T*_m,width_ were inversely correlated to *T*_c_, while no obvious correlations were found between the composites’ microstructural features and thermal properties determined by DSC.

### 3.4. Tensile Tests

**Figure 5:**
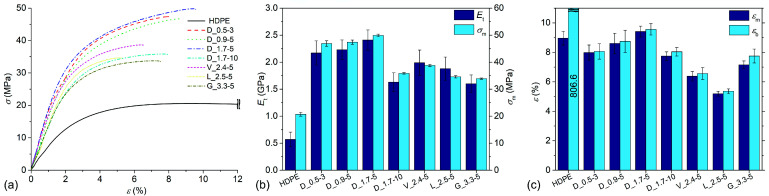
Tensile properties of samples: (**a**) stress–strain curves; (**b**) modulus and strength; (**c**) strain at break and strain at strength.

The tensile properties of the samples are presented in Figure 5. Sample D_1.7-5 exhibited the best overall tensile properties among the composites. In comparison to neat HDPE, the tensile modulus (*E*_t_) increased from 0.57 GPa to 2.41 GPa (+323%) and strength (*σ*_m_) from 20.7 MPa to 49.9 MPa (+141%), while strain at break (*ε*_b_) decreased from 806.6% to 8.8%. The average value of strain at strength (*ε*_m_) was slightly increased (from 9.0% to 9.4%) but without statistical significance (α = 0.05). In comparison to sample D_1.7-5, samples D_0.9-5 and D_0.5-3 had *E*_t_ values in a similar range (2.23 GPa and 2.17 GPa, respectively), while *σ*_m_ was lower at 47.4 MPa (−5%) and 46.9 MPa (−6%), respectively. The difference between samples D_1.7-5 and D_0.9-5 in terms of *ε*_m_ and *ε*_b_ was not significant, while sample D_0.5-3 had lower values of both at 8.0% and 8.1%, respectively. Sample D-1.7-10 performed the worst among the composites containing cylindrical fibres, with *E*_t_, *σ*_m_, *ε*_m_, and *ε*_b_ values of 1.63 GPa, 35.8 MPa, 7.7%, and 8.0%, respectively. In general, non-cylindrical fibres performed much worse in comparison to cylindrical fibres. The *E*_t_ of samples V_2.4-5 and L_2.5-5 was comparable (1.9 GPa and 2.0 GPa), while the *E*_t_ of sample G_3.3-5 was lower (1.6 GPa). Sample V_2.4-5 had the highest *σ*_m_ (38.8 MPa) among the composites containing the non-cylindrical fibres, followed by L_2.5-5 and G_3.3-5 at 34.6 MPa (−11%) and 33.9 MPa (−13%), respectively. In contrast, the latter sample had the highest values of *ε*_m_ (7.2%) and *ε*_b_ (7.8%), followed by V_2.4-5 (6.4% and 6.6%, respectively), and L_2.5-5 (5.2% and 5.4%, respectively). Since the composites were of the same composition but only differed in fibre geometry, it is reasonable to assume that the large majority of the differences between the composites’ mechanical properties originates from the fibre aspect ratio, orientation, and fibre–fibre contact area. In general, a higher fibre aspect ratio and orientation with a lower fibre–fibre contact area result in a composite with higher *E*_t_ and *σ*_m_ values [3,13,14]. It was evident from the microscopic observation of the composites’ microstructures that samples D_1.7-5 and D_1.7-10 had high fibre orientation and a low fibre–fibre contact area. As a consequence, sample D_1.7-5 exhibited the best overall tensile properties, despite samples D_0.9-5 and D_0.5-3 containing fibres with a higher aspect ratio. However, the latter samples had lower fibre orientation and a higher fibre–fibre contact area, resulting in a poor stress transfer to the fibres. In contrast, sample D_1.7-10 exhibited poor tensile properties due to the low aspect ratio of the fibres. The samples containing Viloft^®^ and Leonardo^®^ fibres exhibited slightly higher or similar *E*_t_ and *σ*_m_ values in comparison to sample D_1.7-10 due to the higher aspect ratio of the fibres but also higher fibre–fibre contact area. Sample G_3.3-5 exhibited similar tensile properties to sample D_1.7-10, which is not surprising since both contained severely damaged but relatively well-dispersed fibres. Furthermore, it was observed that the composites containing Viloft^®^ and Leonardo^®^ fibres had values of *E*_t_ and *σ*_m_ in a similar range to, or higher than, samples D_1.7-10 and G_3.3-5 and with lower strains. It appears that a low fibre aspect ratio in combination with a low fibre–fibre surface area results in low *E*_t_ and *σ*_m_ values but comparably high strains, and the opposite combination of the composites’ microstructural features results in contrary effects.

### 3.5. Charpy Impact Tests

**Figure 6:**
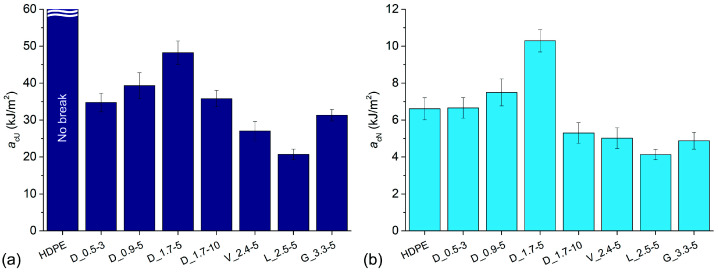
Charpy impact strength of samples: (**a**) unnotched; (**b**) notched.

The Charpy impact strength of unnotched (*a*_cU_) and notched (*a*_cN_) specimens is presented in Figure 6a,b, respectively. The samples of neat HDPE did not break during the unnotched tests, while the *a*_cN_ was 6.6 kJ/m^2^. Sample D_1.7-5 exhibited the highest values of *a*_cU_ (48.2 kJ/m^2^) and *a*_cN_ (10.3 kJ/m^2^) among the composites, where the latter increased by 56% over neat HDPE. In comparison, sample D_0.9-5 exhibited lower *a*_cU_ and *a*_cN_ values of 39.3 kJ/m^2^ (−18%) and 7.5 kJ/m^2^ (−27%), respectively. The Charpy impact strength was even lower for samples D_0.5-3 and D_1.7-10, which exhibited similar values of *a*_cU_ (about 35 kJ/m^2^), whereas the *a*_cN_ of the former (6.7 kJ/m^2^) was higher than that of the latter (5.3 kJ/m^2^). The composites containing non-cylindrical fibres exhibited lower Charpy impact strength than samples containing cylindrical fibres. Sample G_3.3-5 exhibited the highest *a*_cU_ (31.3 kJ/m^2^) of the former composites, followed by samples V_2.4-5 (27.1 kJ/m^2^) and L_2.5-5 (20.7 kJ/m^2^). Samples V_2.4-5 and G_3.3-5 had *a*_cN_ values in the same range (about 5 kJ/m^2^), while the *a*_cN_ was lower (4.1 kJ/m^2^). In general, the Charpy impact strength of fibre-reinforced polymer composites depends on the same factors as *σ*_m_ but to varying degrees [35]. In our study, the *a*_cU_ of composites corresponded very well to *ε*_m_ and *ε*_b_ values (Pearson’s correlation coefficient (*r*) of 0.985 and 0.978, respectively), although with a slightly higher magnitude of effects.

### 3.6. Dynamic Mechanical Properties

**Figure 7:**
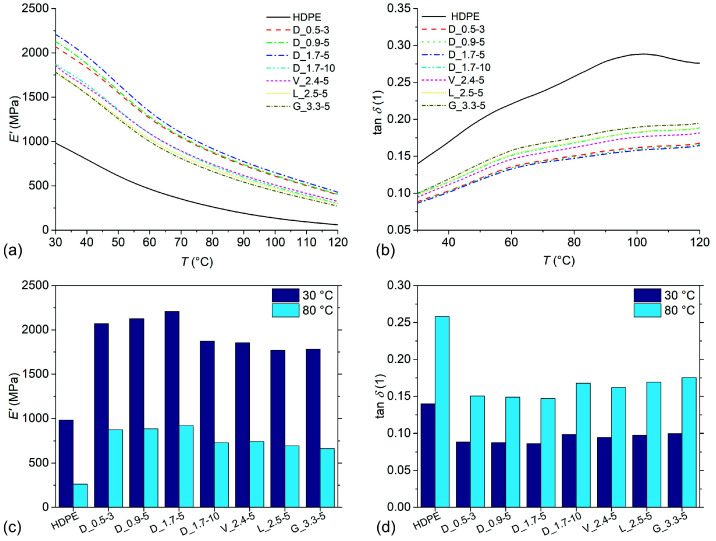
Dynamic mechanical properties of samples: (**a**) storage modulus *E*′ as a function of temperature; (**b**) loss factor tan *δ* as a function of temperature; (**c**) values of storage modulus *E*′ at 30 °C and 80 °C; (**d**) values of loss factor tan *δ* at 30 °C and 80 °C.

The storage modulus (*E*′) and loss factor (tan *δ*) as a function of temperature are presented in Figure 7a,b, respectively. The composites exhibited significantly increased *E*′ and decreased tan *δ* values in comparison to neat HDPE over the whole investigated temperature range. The *E*′ of the samples decreased linearly with increasing temperature up to around 55 °C. At this temperature, a transition region was observed with a decreasing slope that persisted to around 85 °C, above which the *E*′ again decreased linearly. The tan *δ* of the samples increased with increasing temperature, except for neat HDPE where the tan *δ* started to decrease at around 100 °C. At about 60 °C, a tan *δ* peak was observed which was ascribed to the α-relaxation of HDPE [36]. Both the *E*′ transition region and relaxation peak were shifted to slightly higher temperatures for the composites, which were attributed to strong interactions between the matrix and the fibres due to the presence of HDPE-g-MA [37]. The *E*′ values of samples at 30 °C and 80 °C are presented in Figure 7c. Neat HDPE had *E*′ values of 982 MPa and 262 MPa at 30 °C and 80 °C, respectively. Sample D_1.7-5 exhibited the highest values of *E*′ at 30 °C and 80 °C, with values of 2208 MPa (+125%) and 920 Mpa (+251%), respectively. The *E*′ of composites was closely correlated to their *σ*_m_, with *r* coefficients of 0.981 and 0.993 for the *E*′ at 30 °C and 80 °C, respectively. The reinforcing effect of the fibres is especially prominent at elevated temperatures as the *E*′ of the matrix decreases, while the fibres still retain a comparably high level of stiffness. Close correlations between the *E*′ and *σ*_m_ suggest that composites’ microstructural factors have the same influence on the stiffness and strength. While the correlations between the *E*_t_ and *σ*_m_ were relatively high (*r* coefficient of 0.934), the correlations between the *E*′ and *σ*_m_ were significantly higher. This may be explained by the higher modulus measurement precision of DMA in comparison to tensile tests, evidenced by the high measurement standard deviations of the latter technique. The tan *δ* values of the samples at 30 °C and 80 °C are presented in Figure 7d. The tan *δ* values of the composites were inversely correlated to the *E*′ at 80 °C, with *r* coefficients of 0.983 and 0.989 for the tan *δ* at 30 °C and 80 °C, respectively, implying that the damping behaviour of the composites is mostly dependent on the available stress transfer between the matrix and the fibres, while the heat dissipation effects on the matrix/fibre interface have only a minor influence.

## 4. Conclusions

Composites based on HDPE and 30 wt.% of VF with varying geometry were prepared by compounding and injection moulding. The composites with cylindrical fibres with a linear density of 1.7 dtex exhibited the best fibre dispersion, the highest orientation, and the lowest fibre–fibre contact area. Decreasing the linear density of the cylindrical fibres resulted in progressively worse fibre dispersion and orientation. The non-cylindrical fibres were relatively well dispersed and oriented, but their contact area was larger, which was explained by different fibre packing properties. A fibre length analysis revealed that there is an optimal fibre linear density and initial length in terms of preserving their length during processing. Too long or thin fibres tend to entangle, resulting in higher fibre damage during processing. Moreover, fibres with a high cross-sectional aspect ratio are easier to break, while geometries that allow mechanical interlocking between the fibres can lead to additional fibre damage. In general, the composites containing cylindrical fibres exhibited better tensile properties than those with non-cylindrical fibres. The best tensile properties were found for composites with the former type of fibres with a linear density of 1.7 dtex and an initial length of 5 mm. It was found that a low fibre aspect ratio and low fibre–fibre contact area result in a low tensile modulus and strength but comparably high strains. The Charpy impact strength of the composites was highly correlated with strain at strength and strain at break, while the tensile and storage moduli were correlated with tensile strength. Our study provides the first insights into the influence of all the parameters related to the fibre geometry (cross-section shape, linear density, and length) on the resulting microstructure–property relationships of MMCF-reinforced TMCs. The results showed that the proper fibre geometry is of paramount importance to ensure the optimal mechanical properties of the composites. However, the present study has some important limitations that need to be considered. The composites were prepared by compressing the fibres into pellets, which is known to impede fibre dispersion and results in fibre damage. The results could be significantly different if the fibres were processed directly by equipment capable of handling low-bulk-density materials. The influence of the fibre geometry on the structure–property relationships of composites may be highly co-dependent on different processing variables that influence the dispersive mixing capabilities, such as the extruder’s screw geometry and screw speed. Difficult-to-disperse fibres (e.g., thin, high aspect ratio fibres) might perform better under high shear mixing conditions or when using a lower fibre loading.

## Figures and Tables

**Table 1:** Commercial names and geometrical properties of VF and abbreviations of the corresponding composites.

Commercial Name	Cross-Section Shape	Linear Density (dtex)	Length (*L*) (mm)	Diameter/Thickness (µm)	Width (µm)	*D*_EQ_ (µm)	*L*/*D*_EQ_ (1)	Abbreviation of Composite
Danufil^®^	Circular	0.5	3	6.47 ^a^	/	6.47	464	D_0.5-3
Danufil^®^	Circular	0.9	5	8.68 ^a^	/	8.68	576	D_0.9-5
Danufil^®^	Circular	1.7	5	11.93 ^a^	/	11.93	419	D_1.7-5
Danufil^®^	Circular	1.7	10	11.93 ^a^	/	11.93	838	D_1.7-10
Viloft^®^	Rectangular	2.4	5	4 ^b^	40 ^b^	14.18	353	V_2.4-5
Leonardo^®^	Rectangular	2.5	5	3 ^b^	60 ^b^	14.47	346	L_2.5-5
Galaxy^®^	Trilobal	3.3	5	6 ^b^	14 ^c^	16.63	301	G_3.3-5

^a^ Values based on linear density; ^b^ approximate values provided by the manufacturer; ^c^ width of the sides.

**Table 2:** Composition of samples.

Component	Content (wt.%)
HDPE	63.82
VF	30.00
HDPE-g-MA	4.00
Slip agent	1.80
Antioxidant	0.38

**Table 3:** Compounding temperature profile.

Zone	Die	10	9	8	7	6	5	4	3	2	1
Temperature (°C)	165	165	160	160	165	165	160	155	145	140	135

**Table 4:** Injection moulding processing parameters.

Processing Parameter	Values and Units
Barrel temperature—hopper to nozzle	145 °C, 150 °C, 155 °C, 160 °C, 160 °C
Injection velocity profile	50 mm/s, 5 mm/s last 3 mm
Switch-over point	6.5 mm
Packing pressure profile	70 MPa (1 s), 120 MPa (10 s), 30 MPa (2 s)
Metering stroke	20 mm
Decompression	5 mm
Screw angular velocity	175 min^−1^
Backpressure	19.5 MPa
Mould temperature	45 °C
Rest cooling time	9 s

**Table 5:** Thermal properties of samples determined by DSC.

Sample	*T*_c,onset_ (°C)	*T*_c_ (°C)	*T*_c,width_ (K)	Δ*H*_c_ (J/g)	*T*_m_ (°C)	*T*_m,width_ (K)	Δ*H*_m_ (J/g)	*X*_c_ (%)
HDPE	118.0	115.4	7.7	177.5	131.0	9.3	177.5	60.6
D_0.5-3	118.9	112.4	11.8	125.4	134.8	11.5	125.4	63.1
D_0.9-5	118.7	113.1	10.9	121.7	133.8	11.4	121.7	61.3
D_1.7-5	118.7	113.4	10.5	120.7	133.7	10.8	120.7	60.7
D_1.7-10	118.7	112.3	12.1	122.2	135.1	11.2	122.2	61.5
V_2.4-5	118.3	111.2	12.7	121.0	135.6	12.0	121.0	60.9
L_2.5-5	119.1	114.3	10.1	123.9	132.5	10.7	123.9	62.4
G_3.3-5	118.6	113.9	9.7	123.0	132.4	10.5	123.0	61.9

## Data Availability

Not applicable.
